# Inverse pharmacology: Approaches and tools for introducing druggability into engineered proteins

**DOI:** 10.1016/j.biotechadv.2019.107439

**Published:** 2019-12

**Authors:** Jamie A. Davies, Sam Ireland, Simon Harding, Joanna L. Sharman, Christopher Southan, Alazne Dominguez-Monedero

**Affiliations:** a Deanery of Biomedical Sciences, University of Edinburgh, Hugh Robson Building, George Square, Edinburgh EH8 9XB, UK; b Biomolecular Structure & Modelling Unit, Institute of Structural and Molecular Biology, Division of Biosciences, University College London, London WC1E 6BT, UK; c Novo Nordisk Research Centre Oxford, Novo Nordisk Ltd, Innovation Building, Old Road Campus, Roosevelt Drive, Oxford OX3 7FZ, UK; d TW2Informatics, Göteborg 41266, Sweden

**Keywords:** Synthetic biology, Protein engineering, Structure-function, Drug, Pharmaceutical, CRISPR, Cpf1, Gene editing

## Abstract

A major feature of twenty-first century medical research is the development of therapeutic strategies that use ‘biologics’ (large molecules, usually engineered proteins) and living cells instead of, or as well as, the small molecules that were the basis of pharmacology in earlier eras. The high power of these techniques can bring correspondingly high risk, and therefore the need for the potential for external control. One way of exerting control on therapeutic proteins is to make them responsive to small molecules; in a clinical context, these small molecules themselves have to be safe. Conventional pharmacology has resulted in thousands of small molecules licensed for use in humans, and detailed structural data on their binding to their protein targets. In principle, these data can be used to facilitate the engineering of drug-responsive modules, taken from natural proteins, into synthetic proteins. This has been done for some years (for example, Cre-ERT2) but usually in a painstaking manner. Recently, we have developed the bioinformatic tool SynPharm to facilitate the design of drug-responsive proteins. In this review, we outline the history of the field, the design and use of the Synpharm tool, and describe our own experiences in engineering druggability into the Cpf1 effector of CRISPR gene editing.

## 1 Introduction

One of the most striking trends in the development of therapeutics has been a change of focus from the discovery and development of small-molecule drugs (eg aspirin, ranitidine) to the development of large ‘biologics’ (recombinant or engineered proteins, including antibodies, and living cells). The first of these, *Humulin* (insulin made by recombinant DNA technology) was approved by the United States Food and Drug Administration (FDA) in 1982. A further 90 large biologics were approved over the next 30 years (reviewed by Kinch, 2015, Liu et al., 2019).

Biologics have several advantages over small molecule drugs, but also bring some problems. Their main advantage is very high specificity, often coupled with high efficacy. Biologics also tend to benefit from shorter development times than small molecules (especially when targeted against rare diseases) and a lower rate of withdrawal due to safety concerns identified during human clinical trials (reviewed by Kinch, 2015). These large molecules have two main disadvantages. One is that, being large, they are potential targets for immune recognition, which can limit their long-term or repeated use against chronic conditions (Kuriakose et al., 2016): in a recent review of the prescribing information for 121 FDA-approved biological products, Yow-Ming et al. (2016) found that 89% had been reported to stimulate production of anti-drug antibodies and, in 60%, activity-inhibiting antibodies were reported. The other problem arises from their power: some constructs, especially those designed to activate the immune/ inflammatory systems, can run the risk of triggering an excessive response. An infamous example was Theralizumab (TGN1412), an activating antibody against CD28, a receptor that is normally part of the co-stimulation response involved in activating T cells. Theralizumab can activate T cells even in the absence of antigen-derived signals (‘superagonism’); in animal trials it acted preferentially on regulatory T cells and thus dampened immune activation. Its use in humans, however, caused very serious inflammatory reactions in a first-in-human study in 2013, causing long-term harm to volunteers and the bankruptcy of the developing company (Kenter and Cohen, 2006; Stebbings et al., 2009). One response to this has been the improvement of the governance and practice of phase I trials of this type of molecule (reviewed by Tranter et al., 2013), and indeed the development of Theralizumab has continued under other management (Tyrsin et al., 2016). Another important response has been a greater interest in building intrinsic safety and control systems into the biologic therapeutics, mainly at the level of cells but also, in principle at least, at the level of molecules (Straathof et al., 2005; Di Stasi et al., 2011; Minagawa et al., 2015).

Cellular therapies have stimulated researchers to design a variety of externally-controllable ‘kill-switches’, designed either to inhibit the activity of the cells or literally to kill them. Genetic constructs have been built that kill their host cells in response to either the presence or the loss of a specific small molecule. For example, Chan et al. (2016) engineered a strain of *E. coli* that could survive only in the presence of anhydrotetracycline and, in its absence, would switch to a suicidal pattern of gene expression. Some systems have taken careful note of the risk of selection pressure eliminating kill switches from a cell's genome, and have produced systems that are evolutionarily stable, both in theory and in practice, as far as this has been tested (Stirling et al., 2017). A broadly similar approach, in the sense that external control relies on the concentration of a small molecule, has been used to modulate the activity of cells used for cell therapy. An example is the Go-CAR-T version of the Chimaeric Antigen Receptor-T cell (CAR-T) system for activating anti-tumour T-cells without the need for co-stimulation by antigen-presenting cells (reviewed by Feins et al., 2019). In this system, therapeutic engineered T cells contain both an engineered T-cell receptor (TCR), which recognizes a tumour antigen, and an engineered version of the co-stimulation receptor that is activated by a small molecule rather than by an antigen-presenting cell. The maximum activation of the anti-tumour T cells can therefore be controlled externally via the small molecule rimiducid, reducing the risk of an out-of-control hyperactivation of the immune system (Foster et al., 2017).

Controlling the survival or activity of whole therapeutic cells with small molecules is relatively straightforward, as long as the need for genetic engineering of those cells is accepted, because the control elements can be separate from those that perform the cells' therapeutic task. Including such controls in biologics that are based on proteins, rather than on whole cells, is more challenging because of the need to introduce a control element without disrupting the molecules' primary function. Despite the difficulty, such control has been achieved in a few cases, primarily for research purposes. A famous example is Cre-ERT2, which is a chimaera of Cre recombinase and the Tamoxifen-sensitive variant of the Estrogen receptor, ERT2. Cre-ERT2 is active only in the presence of Estrogen (Feil et al., 1996; Zhang et al., 1996), a property that is widely used to activate ‘floxed’ genes only at a specific stage of animal development (reviewed by Zhang et al., 2012, Wilm and Muñoz-Chapuli, 2016). Achievements such as these give hope to the idea that introducing small-molecule control to clinical therapeutic proteins may be feasible, whether these proteins are made in the body following gene therapy or are made outside it and administered as therapeutic biologics.

In the rest of this short review, we will explain a general approach to the design of controllable proteins, outline the features of new bioinformatic tools intended to make the design process easier, and describe a wet-lab demonstration of the idea.

## 2 The concept of ‘inverse pharmacology’

### 2.1 Defining ‘inverse pharmacology’

Traditional pharmacology is a directional process that begins with the identification of a target for example, a component of a cellular signalling pathway such as a protein kinase enzyme. It then involves screening small-molecules for an ability to bind to and modulate the activity of that target. From this set of small molecules, candidate drugs are developed, often by rational modification of the original molecules to improve specificity, efficacy or kinetic parameters. These are then subjected to preclinical and clinical testing, ideally resulting in approved drugs for clinical use. The result of this is a current total of about 1300 small-molecule drugs that are approved for use in humans (European Medicine Agency data, downloaded from *ema.europa.eu*. August 2018).

Research on drug-target interactions, performed both during drug development and post-hoc, has produced high-resolution structural information for a large number of protein targets (Somody et al., 2017), and identification of the amino-acid residues that are involved in interactions between the protein and the drug. These data are available on on-line databases (Chen et al., 2001; Gilson et al., 2016; Desaphy et al., 2015; Liu et al., 2015, Mura et al., 2018, Young et al., 2018). In principle, it should be possible to use these data to identify “drug-binding modules” in natural proteins that might be engineered into chimaeric therapeutic proteins to confer control on them by the same drug. This idea, proceeding from known drug to the design of a novel protein, runs in the opposite direction to the traditional from-known-protein-to-designed-drug approach: for this reason, we call it ‘inverse pharmacology’.

### 2.2 Identifying promising (and unpromising) drug-binding ‘modules’

**Fig. 1:**
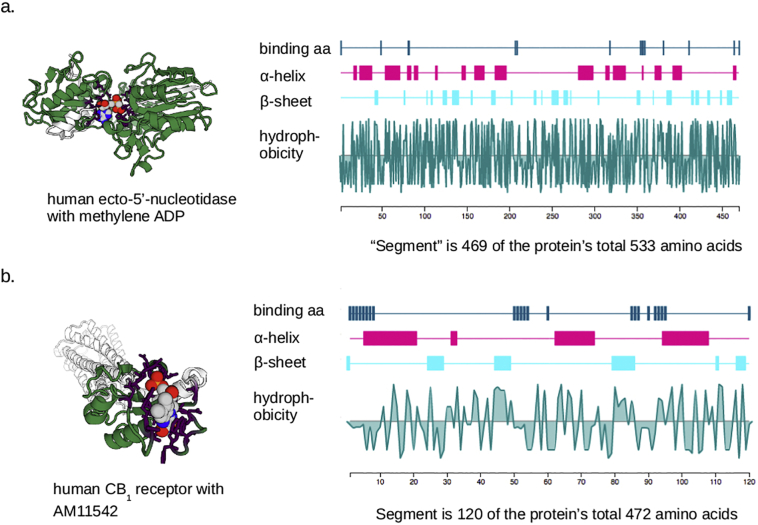
Examples of drug binding sites formed by amino acids spaced widely in a protein, or those in a relatively defined segment. (a) Shows the structure of human ecto-5′-nucleotidase binding to the drug, αβmethylene-ADP. From the first amino acid involved in the binding to the last, the drug-binding segment includes 469 of the protein's 533 amino acids, and the binding pocket is clearly dependent on the way the whole protein folds to locate these amino acids in space. This would not be a promising candidate as a ‘module’ that could be included in engineered proteins. (b) Shows the human CB1 receptor complexed with the drug AM11452. Here the drug-binding segment constitutes only 120 of the protein's 472 amino acids, and the drug-binding site is on the edge of the protein, much less dependent on the protein's overall structure. This is therefore a more promising candidate to be a transferrable module. Note that the plots on the right of the figure show only the drug-binding segment, not the whole protein. These images come from the SynPHARM web tool described in the text.

The binding of a small molecule to a protein depends on the 3-dimensional structures, and surface fractional charge distributions, or both. The 3-dimensional shapes of proteins are dominated by their secondary, tertiary and sometimes even quaternary structures, some aspects of which arise from the primary structure (amino acid sequences) and some from interactions with other proteins such as chaperones during their synthesis (see Englander and Mayne, 2014, for a recent review). The result of this is that some drug-binding pockets arise from the spatial juxtaposition of amino-acid residues that are far apart from one another in the primary sequence, and whose proximity in space is an emergent property of the whole protein. A real example is shown in Fig. 1a. Other drug-binding pockets, however, come from close local folding of part of an amino-acid chain that is only a relatively small part of the whole protein (Fig. 1b). These are much more promising as ‘modules’ that can be removed from their native context and used in an engineered, chimeric, protein to confer the property of binding the drug. For these reasons, the degree to which drug-interacting amino-acids are in a short segment of primary sequence, rather than spread out all through the peptide chain, is a primary selection criterion for finding possible ‘druggable modules’. (See Table 1.)

**Table 1:** Potential applications of the SynPHARM tool.

SynPHARM used to;	To help build;	For this end application;
	Drug-controlled enzymes	Inducible gene editing. Process control/ parameter passing for synthetic biological biosynthesis systems. Rapid modulation of therapeutic enzymes.
Identify drug-binding sites in proteins that might be transferred as modules to other proteins.	Drug-controlled transcription factors or signal receptors	Process control/parameter passing for synthetic biological biosynthesis systems. Modulation of activities of therapeutic engineered cells. ‘Kill-switches’ for therapeutic engineered cells.
	Drug-binding but otherwise inert proteins	Competitive inhibitors of drugs (eg for overdose).
	Drug-modulated protein interactions	Better pharmacologic targeting of protein-protein interactions (which are traditionally hard to target with small molecules).

Once a set of potential modules that pass the above criteria has been identified, further criteria can be applied to rank them in terms of promise. One criterion is the relative independence of the structure of the drug-binding section of the peptide chain from that of the rest of the protein, for example because it is on a projecting fold rather than a fold deep inside the structure. This property is likely to be valuable because a drug-binding module that works as an external fold is more likely to work as an external fold of a completely different, chimaeric protein, without interfering (when there is no drug) with the function of that protein. Another criterion, which depends a lot on the final intended application, is that the drug-binding function can be placed closely enough to a functional group in the final chimaera for the drug to interfere with the function of that group (for example, blocking access to an enzyme or to a heat-shock protein).

### 2.3 Identifying behaviours that drug-binding can modulate

**Fig. 2:**
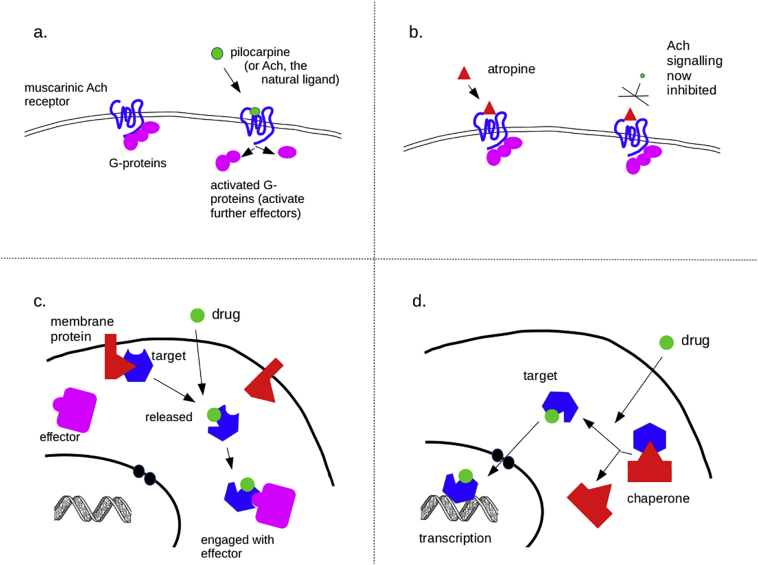
some classic modes of drug action, and their potential for use in engineered systems. (a) Depicts classical agonism, in which a drug (in this case pilocarpine) mimics the action of a natural ligand (in this case, acetylcholine, Ach) on a receptor (in this case, the M3 muscarinic acetylcholine receptor). (b) Depicts antagonism, in which a drug (in this case, atropine, a competitive neutral antagonist) inhibits the action of the natural ligand on the same M3 receptor. It should be noted that other, more subtle, drug actions exist but they are beyond the scope of this article. (c,d) Depict schemes in which a drug displaces a target from a protein, to which it is otherwise bound and sequestered, in a way that prevents the target interacting with its effector, either a cytoplasmic molecule (c) or the genome (d). Because the schemes in (c,d) rely only on modulating a target's binding to a membrane protein or chaperone, their action is relatively independent of the global structure of the target, so might be transferrable to a chimaeric protein. The DNA graphic in (c,d) is from US Department of Energy and is public domain (source: https://commons.wikimedia.org/wiki/File:Dna-split.png).

In conventional pharmacology, most drugs act either as agonists or antagonists according to whether they increase or decrease a target biological process (which is not necessarily the same as whether they increase or decrease the activity of a target molecule). Generally, direct agonists work by mimicking the natural ligand for a receptor, and thus trigger receptor activity, for example causing activation of G proteins. An example would be the cholinomimetic alkaloid, pilocarpine, which mimics acetylcholine and activates muscarinic acetylcholine M_3_ receptors (Fig. 2a). In general, the binding sites for direct agonists are connected closely with the specific function of the target protein, and would be difficult to transfer, in a ‘module’, to an engineered protein with a different function.

Indirect agonists work not by activating their target but by other mechanisms. These include preventing the interaction of a naturally activated target with natural molecules that would inactivate it. An example is the acetylcholinesterase inhibitor neostigmine, which prevents destruction of acetylcholine by acetylcholinesterase and therefore promotes cholinergic signalling. Many antagonists also work by binding to a target in a way that prevents a natural molecule binding to that target. An common example is atropine, which binds to the muscarinic acetylcholine receptors and prevents the natural ligand, acetylcholine, from binding to and activating them (Fig. 2b). This type of drug action, basically getting-in-the-way-of-something, is a much more promising type of interaction for the purposes of transferring it to other molecules.

There are two main ways in which getting-in-the-way can be used. One is steric hindrance, in which the presence of a drug on one part of a peptide blocks the function of an adjacent part of the peptide, for example of an active enzymatic site, either by constraining its range of folding or by interfering with access by natural small molecules. Another approach, more generalisable, is competitive inhibition of protein-protein interactions. For example, an engineered protein may be constructed so that it interacts with a membrane protein, thus trapping it at the membrane (Fig. 2c), or so that it interacts with a heat-shock protein, thus trapping it in the cytoplasm (Fig. 2d). If the drug-binding module is engineered close to the protein-protein binding module, or better still if the drug-binding module already is a protein-protein binding module, then presence of the drug will release the engineered protein and allow it to diffuse to another part of the cell, for example the nucleus to trigger transcription. This was the approach taken by to create Cre-ERT2 (Feil et al., 1996; Zhang et al., 1996).

## 3 Synpharm: a tool to facilitate inverse pharmacology

### 3.1 The need for dedicated informatic tools

Information on protein structures, and on the binding of drugs to proteins, has been available electronically for many years (Young et al., 2018). ChEMBL (Gaulton et al., 2017) has rich data on drug structures; PDB (Bernstein et al., 1977; Berman et al., 2016) holds thousands of protein structures determined by crystallography, NMR and cryo-EM; Guide to PHARMACOLOGY (Harding et al., 2018; Sharman et al., 2018) presents data on drug-target partnerships, and BindingDB (Chen et al., 2001; Gilson et al., 2016) contains quantitative data on interactions between small molecules and proteins, including some structural information. All of these are open and free. The data necessary for making lists of potential ‘druggable modules’, and for ranking them in terms of promise, are therefore fully available. The power of these databases lies partly in their size and scope (ChEMBL, for example, contains information on more than 1,600,000 compounds: Gaulton et al., 2017), but this power also brings a problem for anyone wanting to identify ‘druggable modules’ manually. For this reason, we have recently constructed a new tool, as an adjunct to the Guide to PHARMACOLOGY database (GtoPdb). The tool is designed to retrieve data from the other sites and to present them in a manner useful for identifying potential ‘druggable modules’ for use in the design of synthetic proteins. It is called SynPHARM and is available at synpharm.guidetopharmacology.org/.

### 3.2 Creation of the SynPHARM tool

There are two aspects of the SynPHARM tool suite: Python scripts that run ‘behind the scenes’ to scan GtoPdb and other databases to identify and correctly assemble relevant data, and the web pages and services made available directly to users. The main purpose of the scripts is to abstract relevant entries from large databases ahead of time, with manual quality control, so that online users of the web-based tool can benefit from a high-speed service with curated data when they make their requests. The scripts run at each update of GtoPdb: they identify all drug targets in GtoPdb that have PDB descriptions for ligand-target interactions. Within this list, they identify the amino-acids on the target that mediate interactions with the drug, using a variety of techniques, depending on exactly what binding data are available. Examples in which these amino acids are located on different peptide chains, or in which data are seriously incomplete, are removed from the list. At the last run of these scripts (summer 2018), 618 sequences remained in the list at the end of these processes.

The next stage of processing is the creation of metrics to be associated with each drug-target binding in the list. These metrics are (i) the length of the segment of the peptide sequence containing the amino-acids binding the drug, defined as the segment from the first to last of the drug-interacting amino acids in the whole amino-acid chain, expressed as a proportion of the whole peptide; (ii) the contact ratio (ratio of internal non-hydrogen atom-to-atom interactions within the peptide, and between the peptide and drug, within the segment defined as above).

The data produced by these ‘behind the scenes’ scripts are stored in a PostgreSQL database, connected to a web page using a Java web application, which provides an intuitive interface for users to query the database and view the results in a variety of graphical and text formats.

### 3.3 Use of the SynPHARM tool

**Fig. 3:**
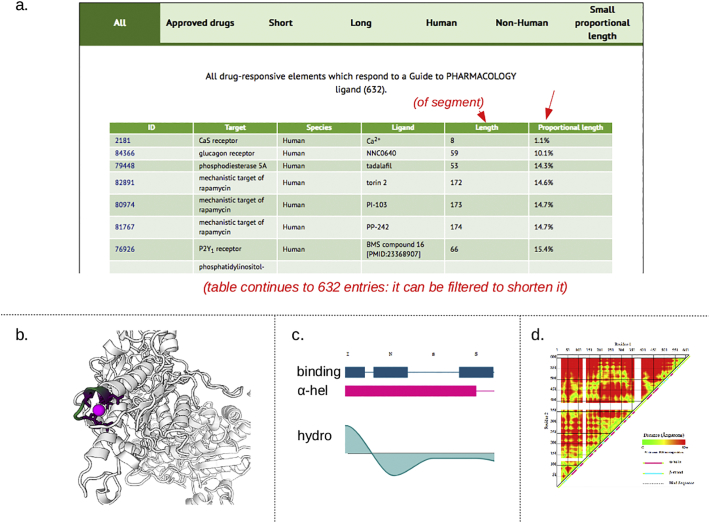
Screenshots from the SynPHARM tool. (a) Shows a small part of a complete table of targets, ordered by ‘proportional length’ of the drug-binding segment compared to the whole molecule. (b,c,d) Depict data on the CaS receptor at the top of the table (viewed by clicking on its index number); (b) Shows a rotatable 3D model, with the drug-binding element coloured, (c) shows positions of drug-interacting amino acids within this segment, and (d) depicts in colour the inter-residue distances in the whole protein. White gaps represent absence of reliable data.

The home page of the SynPHARM tool summarizes the numbers of drugs and targets covered by the tool, subdivided by families, and offers several options for querying the database. It is possible to search for data associated with a specific drug or target, but most users interested in engineering druggability into a protein will be looking for the best drug/ module combination rather than starting with a specific drug in mind. Targets can be searched by family or, most flexibly of all, all targets can be selected and ordered by any of their associated data elements. The data element most likely to be useful is the ‘proportional length’ - the length of the drug-binding sequence (as defined in 3.2 above) divided by the length of the complete protein. Ordering the output of the database by this metric will place at the top of the list those proteins in which drug binding is located in a relatively small part (Fig. 3a).

Clicking on any of the protein names will bring up a page that shows data on their structure in several formats. These include a rotatable 3-dimensional (3D) model of the protein and drug interacting (Fig. 3b), and a graph showing the positions of binding residues, secondary structures (eg alpha helices) and hydrophobicity along a linear representation of the peptide chain (Fig. 3c) and a colour-coded representation of inter-residue distance (Fig. 3d). Used together, these will indicate how feasible it might be to regard the segment containing the drug-binding amino-acids as a ‘module’ that might retain this activity when moved to a different protein. The tool presents that data, but leaves final judgement to human operators (it is intended for use only by researchers familiar with protein biochemistry, and is not intended for completely naive users).

The SynPHARM tool does not contain primary data that are not available elsewhere. The main advantage of using the SynPHARM tool is convenience; it automates the work-flow that is needed to highlight potential druggable modules and show their relationships to the structure of their native protein. It also provides convenient illustrations. It does not attempt to design the manipulations needed for engineering the protein-coding gene itself, because these technologies change quickly anyway, and different laboratories have strong preferences for different techniques according to their existing experience and equipment.

## 4 A practical illustration: engineering drug control into CRISPR effectors

CRISPR-mediated gene editing is widely-used technology for gene editing. Originating in bacteria, the Clustered Regularly Interspaced Short Palindromic Repeats (CRISPR) Type II system uses a combination of endonuclease effectors, such as Cas9, and short guide RNAs (gRNAs), to cut DNA sequences complementary to the gRNA (Ishino et al., 1987; Cho et al., 2013; Kim and Lu, 2019). It can be used in its basic form to create mutations (by cutting target DNA and triggering error-prone repair; correct repairs will be cut again whereas mutations will not as they will no longer match the gRNA. This has allowed researchers to inhibit gene function. Engineering of the Cas9 nuclease has generated versions that have no endonuclease activity (termed dCas9) but that are transcriptional activators or inhibitors (Gilbert et al., 2013) that can be targeted to specific DNA sequences by gRNAs (see Dominguez et al., 2016, for a review). Similarly, fusion of endonuclease-deficient dCas9 to epigenetic modifiers has been used to target epigenetic modification to specific genetic loci (Wang and Qi, 2016).

The CRISPR system has proved very powerful, but some older methods for gene disruption and trascriptional regulation, for example the TetR system (Baron and Bujard, 2000; Atze et al., 2016) and Cre-ERT2 (Feil et al., 1996), while less flexible and involving more work, do have the advantage that they are drug-controllable, allowing gene expression or editing to be done at a time / embryonic stage of a researchers' choosing.

For this reason, we chose to demonstrate the inverse pharmacology idea by producing drug-controllable versions of the Cpf1 CRISPR effector (Dominguez-Monedero et al. 2018). This is not the first time that a CRISPR effector nuclease has been made drug-controllable: Oakes et al. (2016) produced a very effective Cas9-ER chimeric protein, the activity of which is responsible to Estrogen. This was achieved by inserting the estrogen receptor alpha's ligand-binding domain (ER-LBD) into Cas9. In normal ER, in the absence of its ligand, ER-LBD interacts with the cytoplasmic chaperone protein HSP90, and co-chaperones, and this interaction both holds the ER inactive and facilitates binding of the ligand, when it arrives (Fliss et al., 2000). Binding of the ligand then alters the conformation of the ER-LBD, displaces the HSP90 complex, and allows the ER to bind to DNA and activate transcription (Picard, 2006). It has been known for many years that transfer of ER-LBD to other proteins can allow estrogens to modulate the association between these proteins and Heat Shock Protein 90 (HSP90) complexes, and this modulates their availability to interact with other things such as DNA (Picard, 1994). This was the principle on which the Cas9-ER construct of Oakes et al. worked. The finished product was excellent, but its production involved a truly heroic insertion screen, in which they used in vitro transposition using a Mu transposon to insert a restriction endonuclease site at random points in the Cas9 gene, to create a library of modified Cas9 genes that, between them, had more than 70% amino acid sites containing the restriction site. They then inserted an 86-amino acid PDZ domain from alpha-1-syntrophin into these sites, and tested each cas9-PDZ chimaera for remaining Cas9 activity. This work revealed a set of sites at which insertion of their foreign domain was tolerated by Cas9; these sites tended to be, not surprisingly, around flexible loops and near the ends of alpha-helices. They then performed similar insertions with ER-LBD, and found a similar (but smaller) subset of sites at which the ER-LBD would allow Cas9 to retain most of its activity but would trap it in the cytoplasm except in the presence of the Estrogen analogue, Tamoxifen. Because the genetic target of Cas9 is in the nucleus, this effectively conferred Tamoxifen-dependency on the Cas9 action.

The work of Oakes et al. was an excellent proof that Cas9 could be made druggable, but the creation of the functioning molecule involved a great deal of effort. Cfp1 is an alternative CRISPR effector nuclease, broadly similar in action to Cas9 but with some properties that make it more suitable for editing AT-rich regions (reviewed by Fagerlund et al., 2015; Safari et al., 2019), and a druggable version of this would in principle be useful. There is, though, very little sequence alignment between Cas9 and Cpf1 and their 3-dimensional structures are different Gao et al. (2016), so simply placing a druggable module such as ER-LBD in Cpf1 in the same site used for Cas9 is not a viable approach.

**Fig. 4:**
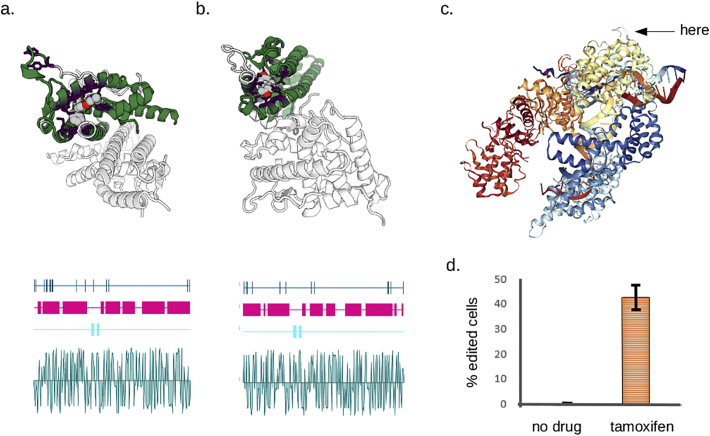
Design of druggable Cpf1. (a) Shows the ligand-binding segment of the human estrogen receptor α, binding tamoxifen; (b) shows the ligand-binding segment of the human progesterone receptor, binding progesterone; (c) depicts the structure of *Acidaminococcus* Cpf1 (PDB entry 5B43, Cpf1 binding to DNA): the position between amino acids 584 and 585, chosen for insertion of the ER-LBD and PR-LBD, is arrowed. (d) Shows the hydroxytamoxifen-dependence of the engineered Cpf1, in an assay in which Cpf1 activity disrupts a repressor and allows expression of a fluorescent reporter: this graph is from Dominguez-Monedero et al., 2018, edited to remove bars referring to other experiments.

We therefore used our bioinformatic tools to examine the structure of ER-LBD and also the progesterone-responsive hPR-LBD (Fig. 4a, b) and Cpf1, and sought to identify a site in Cfp1 that would be likely to tolerate an insertion without destroying Cpf1's activity. The most obvious site was a flexible loop around amino acids 584–585, which protrude from Cpf1 (Fig. 4c). We therefore engineered two chimaeric versions of Cpf1, one with the ERT2 version of ER-LBD in this site, and one with hPR-LBD, in this site (Dominguez-Monedero et al. 2018). Both versions of Cpf1 showed low basal activity, but were activated strongly by their respective ligands (tamoxifen and mifepristone: see for example Fig. 4d). We view this is a preliminary validation of the general approach outlined here.

## 5 Conclusions

Engineering external drug-control into the function of biological therapeutics is a potentially valuable technique for avoiding dangerously strong effects in patients (for example, immune hyperactivation) and it is also useful for research purposes (for example, to activate an activity only at a time of an experimenter's choosing). Given that clinical safety data exist on thousands of approved drugs, it makes sense to make as much use of these as potential regulators as possible (as they avoid the need to test entirely new compounds from scratch): this is ‘inverse pharmacology’. One way to select promising candidates is to screen drug-target binding data, together with data on the structure of the protein target itself, to identify examples of drug-binding ‘modules’ that are likely to retain drug-binding activity even when transferred to engineered proteins. We have produced the SynPHARM bioinformatic tools to facilitate this screening process, and have demonstrated the approach by producing versions of the CRISPR effector Cpf1 that can be controlled by either of two drugs.

Clearly, in conferring steroid control on an endonuclease, we have chosen an especially easy problem, in that we have used well-characterised systems that control nuclear import to control a target protein that cannot work until it is in the nucleus. Exerting strong control on other engineered proteins, that can work in anywhere, may not be as easy but we propose that the inverse pharmacology approach, aided by the SynPHARM tools, will offer the best route towards developing this control.
